# Ultra-Processed Food Intake Is Associated with High Consumption of Added Sugar and Sodium in U.S. Infants

**DOI:** 10.3390/nu18182992

**Published:** 2026-09-13

**Authors:** Kinzie L. Matzeller, Mikayla L. Hernandez, Nathan W. C. Campbell, Edward L. Melanson, Daniel N. Frank, Audrey E. Hendricks, Nancy F. Krebs, Minghua Tang

**Affiliations:** 1 Department of Food Science and Human Nutrition, Colorado State University, Fort Collins, CO 80521, USA; kinzie.matzeller@colostate.edu (K.L.M.);; 2 Department of Pediatrics, Section of Nutrition, University of Colorado Anschutz Medical Campus, Aurora, CO 80045, USA; 3 Department of Medicine, University of Colorado Anschutz Medical Campus, Aurora, CO 80045, USA; 4 Department of Biomedical Informatics, Section of Nutrition, University of Colorado Anschutz Medical Campus, Aurora, CO 80045, USA

**Keywords:** ultra-processed foods, complementary feeding, nutrient intake, infant growth

## Abstract

**Background/Objectives**: Ultra-processed food (UPF) consumption has been connected to adverse outcomes in children and adults, but research in infants is limited. This study assesses the relationship between UPF, dietary intake, and infant growth during complementary feeding. **Methods**: We analyzed infant UPF intake from 3-day diet records at ages 5, 9, and 12 months (*n* = 114). Two coders independently classified foods from diet records using the Nova classification system. Linear mixed-effects models were used to model dietary intake and growth outcomes. A Bonferroni-corrected *p*-value of 0.006 (9 tests) was used for multiple testing. **Results**: UPF intake significantly increased from 9 to 12 months (9 (7, 13)vs. 20 kcal/kg/d (17, 23), *p* < 0.0001), accounting for 30% of total energy at 9 months and 38% at 12 months. UPFs were associated with both added sugar at 9 (β = 0.27 [0.19, 0.36], *p* < 0.0001) and 12 months (β = 0.25 [0.2, 0.3], *p* < 0.0001), and sodium at 9 (β = 16.08 [10.87, 21.29], *p* < 0.0001) and 12 months (β = 12.39 [8.15, 16.64], *p* < 0.0001). Intake of minimally processed foods (Nova 1) was associated with lower added sugar intake (β = −0.22 [−0.27, −0.18], *p* < 0.0001). **Conclusions**: In this cohort of U.S. infants, UPFs had lower nutrient density and higher energy density, with foods such as savory snacks and bars frequently contributing to dietary sodium and added sugar. These findings may suggest that limiting UPFs, especially toddler-specific products, and emphasizing minimally processed foods during complementary feeding could improve adherence to dietary guidance.

## 1. Introduction

With a changing grocery environment in which prepackaged convenience baby foods are increasing in popularity and market share, concerns have emerged regarding the long-term health impacts of infants consuming foods with low nutrient density [1]. Ultra-processed foods (UPF), as one such concern, can stimulate different taste perceptions simultaneously (sweet, salty, and/or umami), driving increased consumption of these products and increasing the potential for nutrient displacement and an unbalanced eating pattern [2]. Given the extensive processing, high palatability, and relatively low nutrient density, UPF during complementary feeding may lower overall infant dietary quality [3]. As such, recent complementary feeding guidelines from the World Health Organization (WHO) recommend avoiding foods high in added sugar, sodium, and trans fats, which are commonly found in UPF [4].

Despite these recommendations from the WHO, United States (U.S.) infants continue to be offered UPF during complementary feeding. A recent estimate suggests that 70% of complementary foods marketed to infants and toddlers in the U.S. are UPFs [5], contributing 45% of daily energy intake between 6 and 23 months of age [1]. Exposure may not be limited to commercial food products, as complementary feeding approaches can often lead to the subsequent provision of adult table foods [6]. U.S. adults consume 55% of their daily energy from UPF, and the general diet of a U.S. adult is often high in sugar and sodium [7,8]. The provision of these adult foods to infants raises concerns about increased sodium and sugar intake in this age group [9]. Due to the high energy density and poor nutrient quality of UPF, it is hypothesized that increased consumption of these foods during complementary feeding can affect dietary intake, growth outcomes, and later risk of obesity [5,10,11,12]. There has been very limited research on UPF intake during complementary feeding, and even less on infant growth trajectories in U.S. infants. This study aims to assess the associations between UPF intake during complementary feeding, nutrient intake, and longitudinal infant growth outcomes using quantitative diet records and repeated growth assessments.

## 2. Materials and Methods

### 2.1. Participants and Sample Size

The current report is a secondary analysis of dietary intakes and anthropometric assessments from 114 infants in the metro Denver area (Colorado, US) (Supplemental Figure S1). Included infants were in the observational groups (reference group) of two randomized controlled trials that recruited participants between 2020 and 2024. Participants in these observational groups did not receive any counseling or dietary interventions and consumed an ad libitum diet. Participants were recruited at 5 months of age by partnering with the Colorado Department of Public Health and Environment to utilize direct mailing. Inclusion criteria were (1) full-term birth; (2) singleton birth; (3) no exposure to antibiotics during delivery or before enrollment; (4) no previous exposure to solid foods (less than one ounce weekly); (5) generally healthy without conditions affecting nutritional status and growth. At ~5 months of age, before participants began complementary feeding, data on maternal age and education, parity, maternal body mass index (BMI), race and ethnicity, gross family income, health history, and feeding history were collected. Three-day diet records for infant dietary intakes, as well as weight and length measurements, were conducted at baseline and repeated at 9 and 12 months of age.

Studies were conducted by the same research team, according to the guidelines laid down in the Declaration of Helsinki, and all procedures involving human subjects were approved by the Colorado Multiple Institutional Review Board. Written consent was obtained from caregivers/legal guardians of all participants. The studies are registered clinicaltrials.gov; NCT04137445 (24 October 2019, Study 1) and NCT05012930 [13] (12 August 2021, Study 2).

### 2.2. Dietary Intake Data

The three-day diet records used to measure dietary intake have demonstrated validity in this age group, compared to estimated energy needs with doubly labeled water and estimated intake with 24 h recalls [14,15]; the same three-day diet records were used for both studies. Caregivers responsible for dietary record completion were educated by the same research team on estimating the amounts of food their infant consumed, including instructions for weighing with a kitchen scale. Bottles of breastmilk or formula were recorded each day, along with duration and frequency of nursing. Caregivers were instructed to, when possible, complete diet records on days when the infant was not in childcare. Because the focus of the present study was on UPFs, liquid diets (breastmilk, infant formula) were excluded from the analysis, keeping only the mode of feeding (breastfed, formula-fed, mixed-fed) as a covariate in the analysis.

Diet records were analyzed by the study team’s registered dietitian (K.L.M.) using the Nutrition Data System for Research (NDSR) software versions 2022–2024, developed by the Nutrition Coordinating Center, University of Minnesota, Minneapolis, MN [16]. For recipes and combination meals, components were manually entered to match published nutrition information when available. For meals without a provided recipe, a generic meal option closest to the identified meal was entered. Branded products were added to match the Nutrition Facts panel available online.

#### 2.2.1. Identifying UPFs

The Nova Classification System for Food Processing (Nova) was used to code individual foods by processing level [3]. Nova was created to evaluate the nature, extent, and purpose of food processing in the relationship between nutrition and disease risk [17], and has been applied previously in toddler and childhood UPF research [1,6,18]. There are four categories of foods in the Nova system, classified by processing level: unprocessed and minimally processed (Nova 1), processed culinary ingredients (Nova 2), processed (Nova 3), and UPFs (Nova 4) [3,19]. The 2019 expansion guidelines of the Nova classification system were used to guide the coding of foods from this cohort [3] (Table 1).

Dietary record data from participants at all three time points contained 1912 unique foods, as shown in Table 1. Two independent coders (K.L.M. and N.W.C.C) assigned a Nova category 1–4 to each unique food identified from the NDSR records; both coders had previous experience in nutrition and dietary recall analysis. Literature review, product labels, and online search engines were used to assign categories. Results from both coders were compared for reliability and reproducibility; ambiguous foods without consensus were reviewed by a third reviewer (M.T.) to assign a final category. Inter-rater agreement was substantial (Cohen’s κ = 0.78 [0.74–0.82]), with conflicting assignment in 53 (3%) of the 1912 foods coded. Intakes of energy and nutrients from each Nova category were assessed as calories per kg (kcal/kg).

#### 2.2.2. Dietary Diversity Assessment

Dietary diversity was also assessed for each infant to evaluate dietary quality with UPF intake. The WHO and United Nations Children’s Fund (UNICEF) Infant and Young Child Feeding Indicators (IYCF) measurement of minimum dietary diversity (MDD) [20] was used. MDD assesses whether children during complementary feeding consume foods and beverages from at least five of eight defined groups. The eight groups include breastmilk; grains, roots, tubers, and plantains; pulses, nuts, and seeds; dairy products (including infant formula); flesh foods; eggs; vitamin-A-rich fruits and vegetables; and other fruits and vegetables. Using the NDSR output of daily consumption, a variety of food groups were combined to form one of the eight groups listed above. For each day of the diet record, the average number of food groups consumed per day was calculated and used to determine if MDD was met (≥5 of 8 food groups) [20].

### 2.3. Weight and Length Measurements

Infant weight and length at 5 and 12 months were measured by pediatric research nurses at the Clinical and Translational Research Center (CTRC) at Children’s Hospital Colorado in triplicate to calculate an average; trained research professionals from the same research team completed anthropometric measurements at 9 months of age. Infant weight was measured using a calibrated Seca^®^ 727 scale to 0.001 kg (Seca^®^, Hamburg, Germany). Length was measured using a calibrated newborn (<70 cm) or pediatric (>70 cm) length board to 0.1 cm (Ellard Instrumentation©, Seattle, WA, U.S.A). Measurements were then used to derive weight-for-age z-scores (WAZ), length-for-age z-scores (LAZ), and weight-for-length z-scores (WLZ) using the WHO growth standards for infants aged 0–24 months [21].

### 2.4. Statistical Analysis

Differences in UPF intake across sample characteristics were assessed using chi-squared tests for binary variables (ethnicity, infant sex, and parity (primiparous vs. multiparous)), and one-way ANOVA for categorical variables (race, maternal education, gross family income category, and maternal BMI category). To assess the association between demographic variables and intake of UPF, linear models controlling for total energy from complementary foods (kcal/kg), study cohort, and mode of feeding were conducted. Associations between UPF intake and outcomes of interest at 5, 9, and 12 months (WAZ, LAZ, WLZ, added sugar and sodium, and average number of food groups consumed) were assessed cross-sectionally using linear models; covariates in linear regression models included total energy (kcal/kg), study cohort, and mode of feeding.

Changes in Nova category intake over time, as well as the relationship between Nova categories and longitudinal growth trajectories (WAZ, LAZ, WLZ), were assessed using linear mixed effects models (lme4, R [22]). As few participants consumed foods from Nova 2 at 5 (*n* = 1), 9 (*n* = 13), and 12 months (*n* = 4), it was combined with Nova 3 for these analyses (Nova 2 & 3). Models evaluated kcal/kg from Nova 1, Nova 2 and 3, and Nova 4 with a random intercept for participant to account for within-subject correlation; covariates included study cohort, total energy, and mode of feeding. Interactions between Nova categories and month were used to assess whether the impact of Nova categories on growth differed over time; significance was assessed at *p* < 0.05 using a likelihood ratio test comparing the full model (with interactions) to the reduced model. Nova categories were compared in pairwise contrasts using the emmeans function using Tukey’s post hoc adjustment. Logistic regression models were used to assess the relationship between UPF intake and odds of meeting MDD, adjusting for study cohort, total energy (kcal/kg), and mode of feeding.

Participants with missing data were excluded case-wise and retained in analyses where data were available. Sensitivity analyses were performed by removing the study cohort to assess model instability due to potential collinearity with mode of feeding, as Study 1 recruited only breastfed infants. To account for multiple testing, a Bonferroni-corrected *p*-value of 0.006 (9 tests [MDD, added sugar, sodium; longitudinal growth (WAZ, LAZ, WLZ) and cross-sectional growth (WAZ, LAZ, WLZ)]) is used throughout the manuscript. Effect sizes and 95% confidence intervals (CI) are provided to facilitate interpretation of results. Statistical analyses were completed in R Statistical Software Version 4.4.2 [23].

## 3. Results

### 3.1. Participant Characteristics

The final sample size was *n* = 114 (Study 1: *n* = 46, Study 2: *n* = 68); a diagram for inclusion of subjects is in Supplemental Figure S1. The demographic characteristics for the total sample are shown in Table 2; as expected, no significant differences in demographic characteristics were observed by study assignment, with the exception of mode of feeding, given that Study 1 recruited only breastfed infants. Sensitivity analyses using linear regression between the Study 1 and Study 2 cohorts revealed no significant differences in UPF intake (β = −1 kcal/kg [−5, 2], *p* = 0.52), calories from complementary foods (β = −14 kcal/kg [−12, 3], *p* = 0.27), or growth between cohorts (WAZ: β = 0.2 [−0.2, 0.6], *p* = 0.29; LAZ: β = 0 [−0.5, 0.5], *p* = 0.97; WLZ: β = 0.3 [−0.1, 0.7], *p* = 0.09). Therefore, results below use pooled data with study assignment as a covariate. Additional sensitivity analyses using nested ANOVA models were performed for covariates including maternal education and pre-pregnancy BMI, gross family income, and race and ethnicity given findings in adults [24,25,26], which did not meaningfully change the estimated associations or improve model fit, and were thus not retained in the final model. Because only complementary foods were included in this UPF analysis, the baseline sample size (5 months of age) was small at *n* = 45, as expected, given the limited number of participants who were introduced to complementary foods at 5 months. Participants with complete dietary intake and growth data at 12 months were not statistically different between studies (Study 1 *n* = 29 (63%), Study 2 *n* = 55 (81%), *p* = 0.06). Descriptive statistics for dietary intake and growth at each timepoint can be found in Supplemental Table S1.

### 3.2. Dietary Intake

#### 3.2.1. Nova Classification of Foods

In total, 30% of the foods classified were unprocessed or minimally processed (Nova 1), 1% were processed culinary ingredients (Nova 2), 28% were classified as processed (Nova 3), and 41% fell into the UPF category (Nova 4). Counts of foods in each Nova category found in infant diet records can be found in Table 1. Linear regression models were used to evaluate differences in nutrient content across Nova categories per 100 g of each food, with pairwise comparisons for estimated means. Considering the average nutrient quality of foods, minimally processed (Nova 1) and UPF (Nova 4) differed. UPF had a higher average energy density (calories per 100 g of food) compared to minimally processed foods (282 kcal/100 g [268, 297] vs. 128 kcal/100 g [112, 145], *p* < 0.0001). UPF had higher sodium than minimally processed foods (326 mg/100 g [297, 354] vs. 219 mg/100 g [185, 252], *p* < 0.0001) after controlling for energy density. Added sugar was not statistically different in UPF after adjusting for multiple testing (5.5 g/100 g [4.5, 6.6] vs. 3.5 g/100 g [2.3, 4.7], *p* = 0.02), though the difference may have clinical significance. Lastly, average fiber was significantly lower in UPF than minimally processed foods (2.2 g/100 g [1.8, 2.6] vs. 3.2 g/100 g [2.8, 3.6], *p* = 0.001).

#### 3.2.2. Total Consumption of UPF

**Figure 1:**
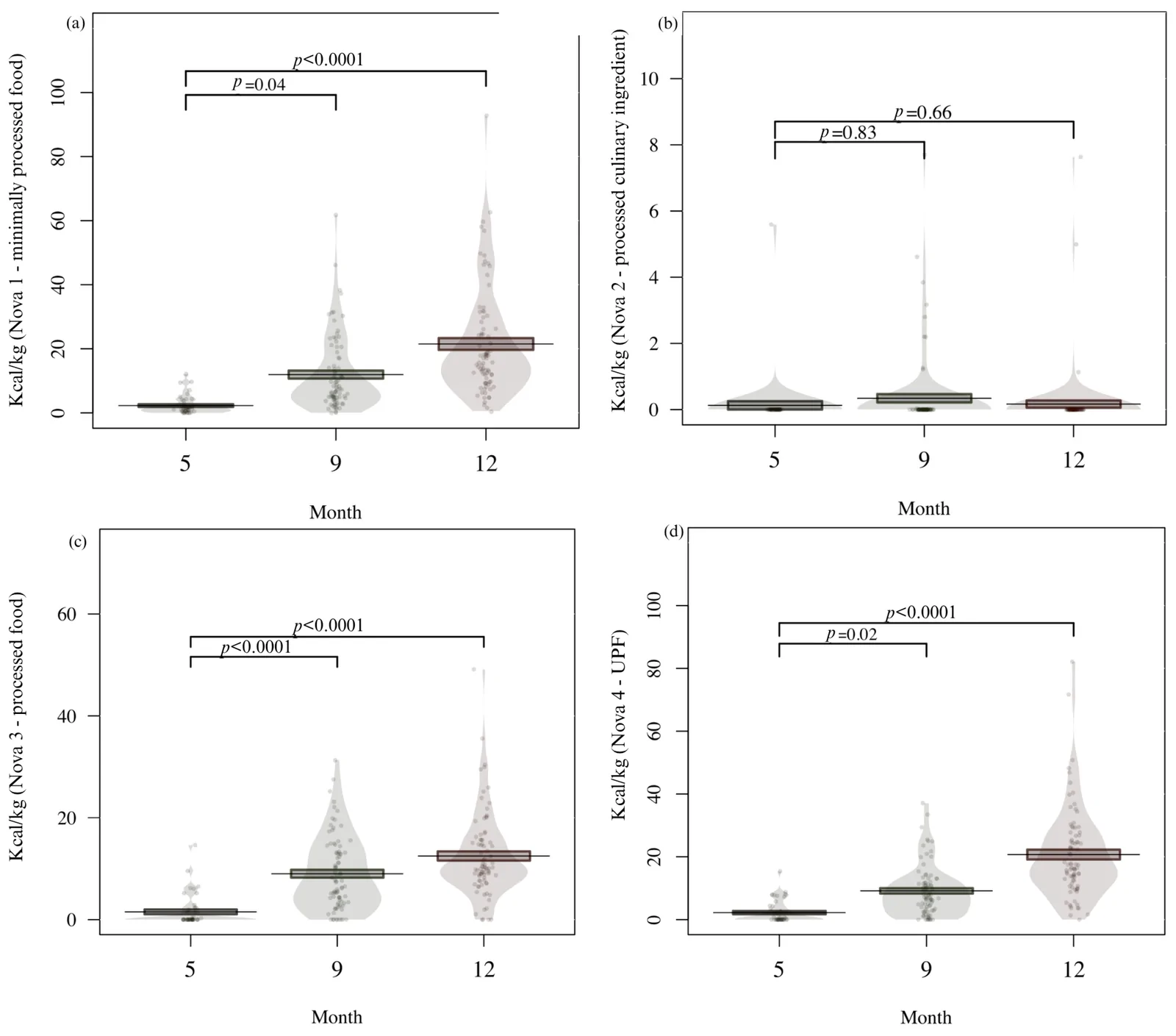
Average intake of foods from Nova categories 1, 3, and 4 significantly increased over time (kcal/kg/d) between 5, 9, and 12 months. Dots represent individual participant observations, black horizontal lines represent group means, boxes represent the standard error around the mean, and shaded areas represent the density of observations. Sample sizes for dietary intake: 5 months (*n* = 45); 9 months (*n* = 85); 12 months (*n* = 84). Shown are (**a**) Nova 1—minimally processed foods; (**b**) Nova 2—processed culinary ingredients; (**c**) Nova 3—processed foods; (**d**) Nova 4—ultra-processed food (UPF). Changes in Nova category intake over time were assessed using linear mixed effects models with total caloric intake, mode of feeding, and study cohort as covariates and included a random intercept for participant to account for within-subject correlation over time; *p*-values for differences in intake using pairwise comparisons are shown. Significance was set at *p* < 0.006 after Bonferroni’s correction.

Consumption of UPF (presented as kcal/kg/d) significantly increased over time, after controlling for increased total energy intake from complementary foods (Figure 1d); the adjusted mean intake of UPF was significantly higher at 12 months (20 kcal/kg [95% confidence interval (CI): 17, 23]) compared to 5 months (4 kcal/kg [1, 8], *p* < 0.001), though not statistically higher at 9 months compared to 5 months (9 kcal/kg [7, 13], *p* = 0.02). Nova 1 (minimally processed) and Nova 3 (processed) foods also increased from 5 to 12 months (β = 12 kcal/kg [6, 19], *p* < 0.0001 and β = 8 kcal/kg [4, 12], *p* < 0.0001). Infants’ consumption of UPF as a percent of energy intake from complementary foods did not change over time after adjusting for total energy intake, study cohort, and mode of feeding (Supplemental Figure S2d).

#### 3.2.3. UPF and Intake of Added Sugar and Sodium

**Figure 2:**
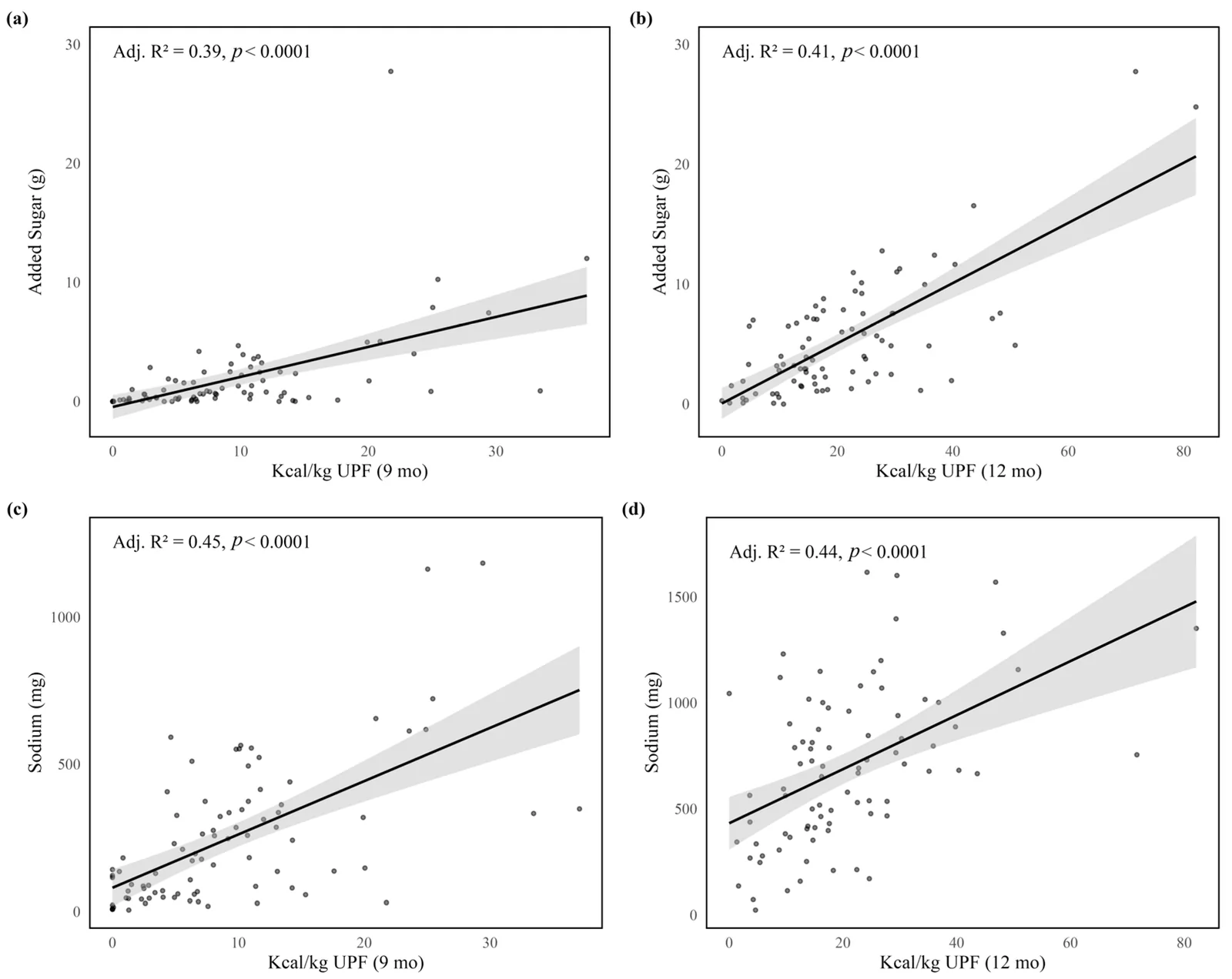
Added sugar intake and sodium intake were significantly associated with UPF consumption measured as kcal/kg/d at 9 and 12 months. Dots represent individual participant observations. Shaded areas represent the 95% confidence interval of the estimated mean response. Sample sizes for dietary intake: 9 months (*n* = 85); 12 months (*n* = 84). Scatterplots of regression models with 95% confidence interval for added sugar intake (non-transformed) and sodium intake given UPF consumption at 9 and 12 months of age. Total kcal/kg/d, mode of feeding, and study cohort were included as covariates in the model. Depicted are (**a**) added sugar (g) by kcal/kg/d UPF at 9 months; (**b**) added sugar (g) by kcal/kg/d UPF at 12 months; (**c**) sodium (mg) by kcal/kg/d UPF at 9 months; (**d**) sodium (mg) by kcal/kg/d UPF at 12 months. R2 and *p*-values for the β estimate are presented for each scatterplot. UPF: ultra-processed food. Significance was set at *p* < 0.006 after Bonferroni’s correction.

Intake of UPF was significantly associated with higher added sugar consumption at both 9 months (Figure 2a, β = 0.27 [0.19, 0.36], *p* < 0.001) and 12 months (Figure 2b, β = 0.25 [0.20, 0.30], *p* < 0.001). Because added sugar intake was not normally distributed, intake at 9 and 12 months was natural log + 1 transformed; associations remained significant after transformation at 9 and 12 months (Supplemental Figure S3). Similarly, an increase in sodium was associated with increased UPF at both 9 months (Figure 2c, β = 16.08 [10.87, 21.29], *p* < 0.001) and 12 months (Figure 2d, β = 12.39 [8.15, 16.64], *p* < 0.001).

Similar models were fit to assess correlations between added sugar and sodium intake and the remaining Nova categories. Higher intake of Nova 1 (β = −0.22 [−0.27, −0.18], *p* < 0.0001) and Nova 2 and 3 (β = −0.22 [−0.28, −0.16], *p* < 0.001) were negatively associated with added sugar intake over time. No significant associations were found between Nova 1 (β = −3.20 [−6.68, 0.27], *p* = 0.07) and Nova 2 and 3 (β = −0.34 [−5.02, 4.34], *p* = 0.89) and sodium intake.

#### 3.2.4. Dietary Diversity

**Figure 3:**
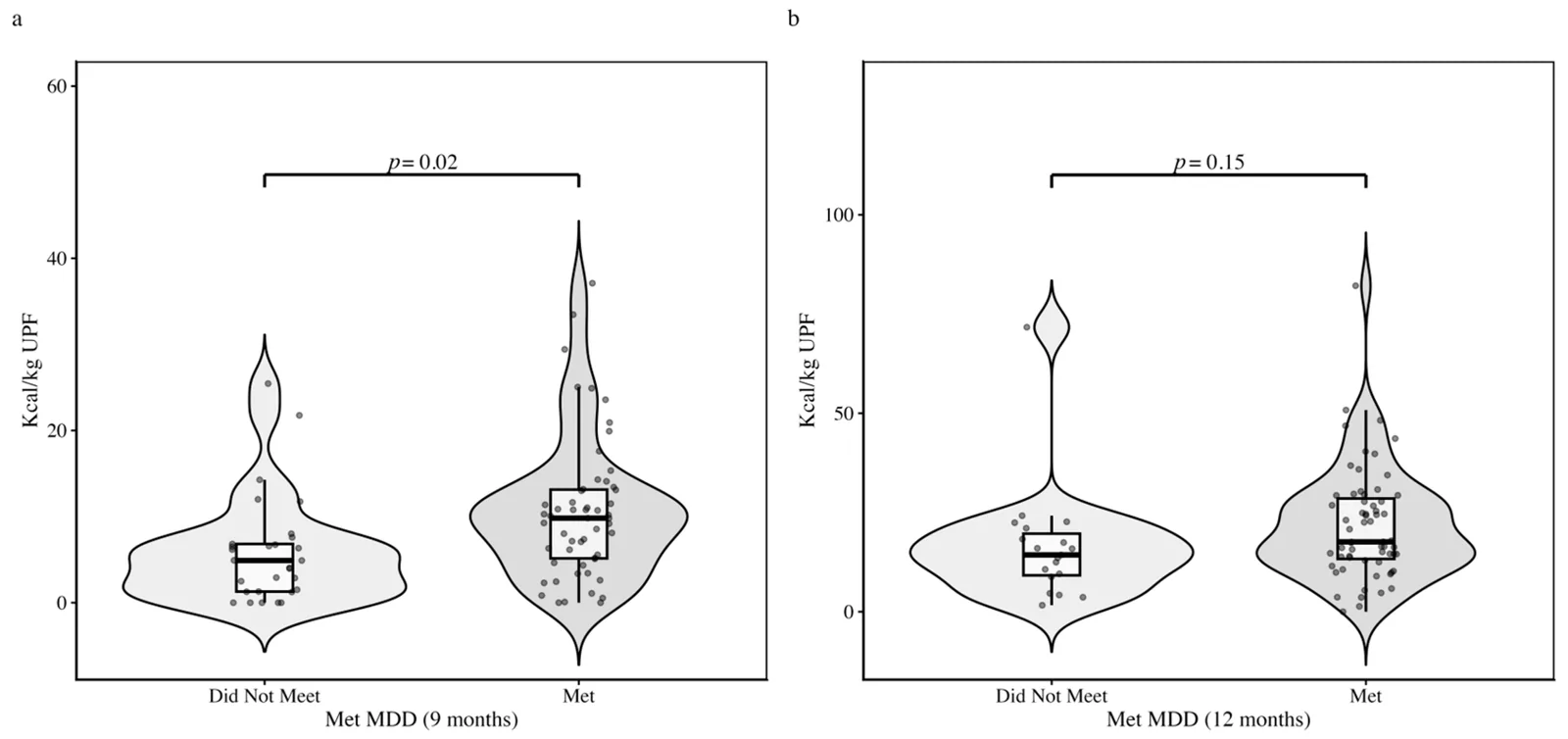
Infants who met Minimum Dietary Diversity (MDD) at 12 months had a higher UPF intake as kcal/kg/day. Dots represent participant observations, shaded areas represent density of observations, black horizontal lines represent group means, and boxes represent the standard error around the mean. Sample sizes for dietary intake were 9 months (*n* = 85); 12 months (*n* = 84). UPF intake, measured as kcal/kg/d, for infants who did and did not meet MDD at 9 and 12 months. Logistic regression included total kcal/kg/d as a covariate in the model. Shown are (**a**) kcal/kg/d from Nova 4 foods at 9 months between infants meeting and not meeting MDD; and (**b**) kcal/kg/d from Nova 4 foods at 12 months between infants meeting and not meeting MDD. Total kcal/kg/d, mode of feeding, and study cohort included as covariates in the model. UPF: ultra-processed food; MDD: minimum dietary diversity. After applying Bonferroni’s correction, significance was set at *p* < 0.006.

Dietary diversity was assessed both as a continuous variable (number of food groups consumed daily) and as a binary variable (MDD met, ≥5 of 8 food groups [20]. No significant associations were found between UPF intake and the average number of food groups consumed daily (β = 0.03 [0, 0.7], *p* = 0.09). When adjusted mean kcal/kg from UPF was compared between infants who did and did not meet MDD, infants who met the MDD tended to consume more UPF (10 kcal/kg (8, 12) vs. 5 kcal/kg (2, 9), *p* = 0.01) than infants who did not meet the MDD at 9 months (Figure 3a). A similar trend was observed at 12 months, which did not meet statistical significance (Figure 3b) (23 vs. 18 kcal/kg, *p* = 0.17). Associations between higher UPF intake and odds of meeting dietary diversity did not meet statistical significance after multiple testing correction at either 9 (odds ratio (OR): 1.15 [1.04, 1.31], *p* = 0.01) or 12 months (odds ratio (OR): 1.03 [0.99, 1.09], *p* = 0.15). Nova 1 and Nova 2 and 3 were not associated with dietary diversity at either timepoint.

Considering mode of feeding and dietary diversity, provision of formula (formula and mixed fed) compared to only breastmilk was associated with providing fewer food groups at 9 months (4.8 groups [4.3, 5.2] vs. 5.8 [5.4, 6.1], respectively *p* = 0.002); differences observed at 12 months between infants receiving formula versus only breastmilk were not significant (5.3 groups [4.9, 5.7] vs. 6 [5.7, 6.3], respectively *p* = 0.06). Results in the sensitivity analysis with Study 2 participants only were consistent between infants receiving any formula versus breastmilk at both 9 and 12 months.

### 3.3. Growth

Linear mixed-effects models were used to assess longitudinal growth trajectories and UPF intake (Table 3). Intake of Nova categories was scaled by 10-unit increments for interpretation; therefore, beta coefficients represent the change in outcome per 10 kcal/kg/d increase. A significant UPF × time interaction was observed for WAZ (*p* < 0.05), indicating that the relationship between UPF intake and WAZ varied over time. At 5 months, greater energy intake from UPF was associated with a lower WAZ (β = −0.52, [−0.93, −0.11]), whereas no significant relationship was observed at 9 (β = 0.00, 95% CI: −0.10, 0.10) or 12 months (β = 0.00, 95% CI: −0.10, 0.10). Findings were robust to the removal of study cohort in sensitivity analyses. No significant interaction was observed for LAZ or WLZ. The associations between UPF intake and WAZ, LAZ, and WLZ were evaluated cross-sectionally at each timepoint using linear regression models, with no significant associations (Supplemental Table S3).

## 4. Discussion

In this study of healthy, term infants from Denver, Colorado, the extent of food processing classified using Nova was associated with intakes of added sugar, sodium, and dietary diversity, but not growth between 5 and 12 months. Infants in this study consumed an average of 32% (26–38%) of their total daily energy from UPFs, which is lower than a previous report using NHANES data, in which 6–11-month-old infants consumed 45% of total energy from UPFs [1]. NHANES collects dietary data using 24 h recalls and is a nationally representative sample [27], compared to the current study from Denver, Colorado, which may explain in part the lower UPF intake. In this analysis, the average percent of energy from each Nova category, including UPF, did not significantly change over time. However, when expressed as kcal/kg/d, all categories except Nova 2 increased; this suggests that minimally processed foods and UPF contributed similar proportions of the diet throughout complementary feeding, while the absolute amount consumed increased as infants transitioned away from a predominantly liquid diet.

Early introduction of added sugar has been associated with a variety of poor health outcomes, including dental caries, asthma, poor diet quality, and overweight and obesity [28]. To our knowledge, this is the first report that UPF intake in U.S. infants is associated with added sugar intake during complementary feeding, despite the lack of nutrient consideration in the UPF definition used in these analyses [19]. This observation aligns with results seen in other age groups; in adults, UPFs are often the main source of added sugar in the diet [29] and are associated with total and free sugar intake in preschool-aged children [30,31]. Examples of commercial products with added sugar consumed by the infants in this cohort include snack bars, instant oatmeal, yogurt melts, and infant mixed meals. Added sugar content of commercial infant and toddler foods has been described previously; most commercial infant foods (marketed for children < 12 months) do not have added sugar, but more than 70% of toddler meals, bars, and snacks (for children ≥ 12 months) contained added sugar [32]. UPF toddler foods consumed by infants in this cohort that contributed added sugar include snack bars, crackers and grain-based savory snacks, pouches, and toddler meals. These findings suggest that UPFs during complementary feeding, compared to less processed options, may be associated with infant added sugar intake; the most recent iterations of the Dietary Guidelines for Americans (2025 and 2030 versions) recommend avoiding added sugar in children under 2 years of age [33,34], suggesting clinical significance of a relationship between UPFs and added sugar.

While sodium is a required nutrient for growth and development, most global country-specific dietary guidelines do not recommend adding salt to baby foods, and consensus is lacking on the appropriate upper limit of sodium for infants during complementary feeding. In this cohort, higher UPF intake was associated with higher dietary sodium. This association may have clinical significance; each 10 kcal/kg increase in UPF intake was associated with a 160 mg/day higher dietary sodium, indicating that UPF may be meaningfully contributing to total sodium exposure during complementary feeding. Furthermore, the WHO recommends avoiding foods high in salt during complementary feeding [4], and the SACN recommends an intake of less than 1 g of salt daily (~400 g of sodium) under 12 months [35]. Most infant foods contain little sodium, with the exception of infant savory snacks and meals [32]. However, as toddler foods have significantly higher sodium [32], avoidance of toddler-specific products in infants younger than 12 months may reduce the likelihood of excessive sodium intake. Additionally, baby-led weaning as a complementary feeding approach has gained popularity in recent decades [36]. Evidence suggests that baby-led weaning may be associated with increased sodium consumption [9,37,38], which may be due to the provision of family foods to infants that are higher in sodium, given the typical U.S. dietary pattern [39,40]. Foods that contributed the most sodium in this cohort were family foods prepared with salt, cured meats, cheese and cheese dishes, and toddler products; infant products contributed little sodium. As less-processed foods in this cohort were not associated with sodium intake, future work should assess persistence of the association between UPFs and sodium into toddlerhood, evaluating longitudinal health outcomes to further understand the implications observed in the current analysis.

At 9 months, infants who met the MDD (≥5 out of 8 food groups) consumed more UPF than those not meeting MDD, a trend that persisted at 12 months. These findings suggest that UPF intake may be associated with dietary diversity during the complementary feeding period; however, UPFs have been shown to negatively affect the nutritional quality of diets in adults, particularly through decreased consumption of fiber, zinc, and fat-soluble vitamins and increased consumption of free sugars [41]. UPFs in this cohort had higher sodium and added sugar, and lower fiber content, despite contributing to overall food group exposure. Examples of foods that provided multiple food groups while contributing to added sugar and sodium intake included infant yogurt blends and toddler meals. The literature also supports that eating patterns, including UPF intake, during the first 2 years of life may persist into later childhood [11,31]. Therefore, while UPFs have the potential to encourage greater dietary diversity during complementary feeding, diet quality and establishment of long-term eating habits should also be considered when selecting complementary foods [42].

Growth outcomes, including weight and length, were not associated with UPF intake in this cohort at 9 or 12 months. In longitudinal models, UPF exhibited a time-varying relationship with WAZ; at baseline (5–6 months), higher UPF intake was associated with a significantly lower WAZ, but this observation did not persist as infants reached 9 and 12 months. The observed relationship may reflect the transitional nature of complementary feeding; at this age, infants should primarily meet their nutrient needs through a liquid diet, which may be displaced by higher UPF intake. Additionally, there is plausibility of reverse causality—infants with a smaller WAZ may have been introduced to complementary foods earlier or given different types of foods in response to growth concerns [43]. It is also possible that this relationship may be a random effect due to the small sample size at baseline (5–6 months), and smaller amounts of foods consumed. No relationship was observed with LAZ. Few studies have reported LAZ outcomes with UPF intake in this age group; however, animal models suggest a possible relationship with bone quality, which may be a target in future work [44]. While evidence of a relationship between UPF and growth in this life stage is limited, previous reports describe a potential relationship between increased intake and overweight and obesity risk, which may be attributed to a lower UPF intake in the current cohort than the average reported by Neri et al. [12]. Additional research in a larger sample size may better capture the true relationship between UPF and infant growth.

The current study has many strengths. Infant dietary intake was assessed by quantitative 3-day diet records, providing a more accurate estimate of usual intake than single-day collection, diet diversity alone, or 24 h recalls [45]. Anthropometric measurements were obtained by pediatric research nurses and trained researchers at each timepoint, and measures were standardized using WHO z-scores, which control for age and sex. Furthermore, infants were followed at three time points during complementary feeding; to our knowledge, this is the first report of longitudinal repeated UPF intake assessment in U.S. infants aged 5–12 months. This study reports novel findings of a positive association between UPF intake during complementary feeding and the intake of nutrients that are generally recommended to be limited during this life stage, such as sodium and added sugar.

While this study provides important information for infant complementary feeding practices, there are several limitations. The overall sample size was modest. The baseline sample size for dietary intake was 45, given the limited number of participants who had introduced solid foods at this timepoint. Also, the Nova classification system was designed to assign categories of processing to foods. However, many infant and baby foods do not align perfectly with the guidelines, which may impact the findings presented here. Despite this, two researchers coded Nova categories for foods with 97% agreement. Foods that were classified differently primarily consisted of mixed meals with unknown variables, such as burritos or pasta (e.g., fettuccine alfredo, spaghetti and meatballs). The current analysis only includes complementary foods and does not include breastmilk or formula intake, and mode of feeding was used as a covariate in the analyses [41,42,43]. However, as total energy intake was not quantified and this analysis only includes complementary foods, we cannot exclude the potential influence of the liquid diet beyond mode of feeding on current findings. Lastly, dietary patterns reported are representative of free-living infants in the Denver, Colorado metro area. As the current study was done in healthy U.S. infants, these results may not be generalizable to other populations without further investigation.

## 5. Conclusions

The current study found that UPF intake during complementary feeding was associated with higher intake of added sugar and sodium in 9- and 12-month-old infants, while less processed foods were associated with lower added sugar intake and had no relationship with sodium. While higher UPF intake was associated with meeting MDD, future work should evaluate whether UPFs increase long-term programming of food preferences alongside nutrient intake and dietary quality. Although no associations were found with growth outcomes, long-term impacts of dietary choices, including UPF intakes, on infant growth, including obesity risk, should be considered in future research. Additionally, further evaluation of the relationship between dietary patterns, food choices, and growth trajectories during complementary feeding may help understand long-term outcomes, including obesity risk and the persistence of eating behaviors. The current findings, along with subsequent research, may then inform guidance on complementary feeding to improve dietary quality and further our understanding of the growth implications of dietary choices during this critical developmental window.

## Figures and Tables

**Table 1:** Number of foods per Nova category identified in this cohort.

Nova Category *	Number of Foods †
1—Unprocessed and minimally processed foods	572 (30%)
2—Processed culinary ingredients	20 (1%)
3—Processed foods	532 (28%)
4—Ultra-processed foods (UPF)	788 (41%)

* Foods from 5, 9, and 12-month diet records were independently coded, totaling *n* = 1912 unique foods. † Counts for each Nova processing category for foods found in infant diet records are presented as *n* (% total).

**Table 2:** Study population characteristics at 5 months (baseline).

Characteristic	*N* (%) *
Infant sex (Female)	60 (53%)
Race	
Asian	4 (4%)
Black/African American	4 (4%)
White	98 (82%)
More than one race	8 (7%)
Ethnicity (Hispanic)	16 (14%)
Mode of feeding	
Exclusively breastfed	87 (76%)
Exclusively formula fed	14 (12%)
Mixed fed	13 (12%)
Parity (Primiparous)	50 (44%)
Maternal pre-pregnancy BMI (kg/m^2^)	
*<18.5*	3 (3%)
*18.5–24.9*	74 (65%)
*25–29.9*	21 (18%)
*>30*	16 (14%)
Maternal education (*n* = 113) †	
Less than a bachelor’s degree	16 (14%)
Bachelor’s degree	44 (39%)
Master’s degree	42 (37%)
Doctorate/Professional degree	11 (10%)
Gross Family Income (*n* = 113) ‡	
<$50,000	3(3%)
$50,000–$150,000	46 (41%)
>$150,000	65 (56%)

BMI, body mass index. * Data presented as *n* (% of total, *n* = 114). † One missing report of maternal education level (Study 2) (*n* = 113). ‡ One missing report of gross family income (Study 1) (*n* = 113).

**Table 3:** Growth outcomes were not significantly associated with UPF intake in longitudinal mixed effects models.

	Model 1: (Base kcal/kg/d) *			Model 2: (Base kcal/kg/d) + Interaction Term †			
	Estimate	95% CI	*p*-Value	Estimate	95% CI	*p*-Value ‡	
WAZ							
9-month timepoint (5-month ref)	0.37	[0.31, 0.43]	<0.0001	0.17	[−0.01, 0.39]	0.13	
12-month timepoint (5-month ref)	0.49	[0.42, 0.56]	<0.0001	0.30	[0.02, 0.58]	0.03	
Nova 1 §	−0.01	[−0.03, 0.01]	0.42	−0.02	[−0.44, 0.43]	0.99	
Nova 2 & 3 §	−0.02	[−0.06, 0.01]	0.21	−0.37	[−0.66, 0.17]	0.25	
Nova 4 §	−0.01	[−0.04, 0.01]	0.26	−0.52	[−0.93, −0.11]	0.001	
9 month: kcal Nova 4 (interaction) §				0.53	[0.09, 0.96]	0.02	
12 month: kcal Nova 4 (interaction) §				0.53	[0.10, 0.95]	0.01	
Total energy intake	0.01	[−0.01, 0.02]	0.22	0.01	[−0.01, 0.02]		0.21
Study cohort (Study 1 ref)	0.14	[−0.29, 0.56]	0.53	0.14	[−0.28, 0.57]	0.50	
Mode of feeding (EBF ref)	0.16	[0.08, 0.24]	0.0001	0.18	[0.01, 0.36]	0.04	
LAZ							
9 month timepoint (5 month ref)	−0.01	[−0.10, 0.08]	0.82				
12 month timepoint (5 month ref)	0.12	[0.02, 0.23]	0.02				
Nova 1 §	0.01	[−0.03, 0.04]	0.79				
Nova 2 & 3 §	0.00	[−0.06, 0.05]	0.90				
Nova 4 §	−0.01	[−0.05, 0.03]	0.51				
Total energy intake	0.01	[-0.01, 0.02]	0.71				
Study cohort (Study 1 ref)	−0.06	[−0.52, 0.40]	0.79				
Mode of feeding (EBF ref)	0.13	[0.01, 0.26]	0.03				
WLZ							
9 month timepoint (5 month ref)	0.41	[0.32, 0.51]	<0.0001				
12 month timepoint (5 month ref)	0.38	[0.28, 0.49]	<0.0001				
Nova 1 §	−0.01	[−0.05, 0.02]	0.34				
Nova 2 & 3 §	−0.03	[−0.09, 0.03]	0.29				
Nova 4 §	−0.01	[−0.05, 0.03]	0.64				
Total energy intake	0.00	[−0.01, 0.02]	0.59				
Study cohort (Study 1 ref)	0.24	[−0.13, 0.61]	0.20				
Mode of feeding (EBF ref)	0.10	[−0.02, 0.22]	0.11				

UPF, ultra-processed food; CI, confidence interval; WAZ, weight-for-age z-score; EBF, exclusively breastfed; LAZ, length-for-age z-score; WLZ, weight-for-length z-score. * Model 1 reflects the base model, including timepoint, Nova 1, 2 & 3, and 4 as kcal/kg/d, total calorie intake, study cohort, and mode of feeding. Nova 2 & 3 were combined for these analyses due to low consumption from Nova 2. Reference category (where applicable) denoted in first column. Sample sizes for dietary intake: 5 months (*n* = 45); 9 months (*n* = 85); 12 months (*n* = 84). † Model 2 includes an interaction term for Nova 4 (kcal/kg/d) by timepoint for WAZ. No interaction term was included for LAZ or WLZ outcomes, as the term was not significant. Nova 2 & 3 were combined for these analyses due to low consumption from Nova 2. Reference category (where applicable) denoted in first column. ‡ For all *p*-values, except interaction terms (*p* < 0.05), significance was set at *p* < 0.006 after applying Bonferroni correction for multiple testing. § In Model 1 and 2, intake of kcal/kg/d was scaled in 10-unit increments, representing the estimated change in outcome per 10 kcal/kg/d increase. Gray cells indicate that no estimate was calculated per model constraints.

## Data Availability

“Restrictions apply to the datasets” as the dataset includes primary outcome data not yet reported, with additional analyses ongoing. We intend to make the appropriate de-identified data available in a public repository once the primary outcomes and planned analyses have been completed.

## Supplementary Materials

The following supporting information can be downloaded at: https://www.mdpi.com/article/10.3390/nu18182992/s1, Table S1: Average dietary intake and growth parameters at 5, 9, and 12 months; Table S2: Growth outcomes were not significantly associated with UPF intake as percent of total energy in longitudinal mixed effects models; Table S3: Dietary intake of UPF was not significantly associated with growth parameters; Figure S1: Subject Flowchart of Analytic Sample; Figure S2: Average intake of foods by Nova category as a percent of total energy consumed did not significantly differ at 5, 9, and 12 months; Figure S3: Natural Log + 1 transformed added sugar intake (g) was significantly associated with consumption (kcal/kg/d) of ultra-processed foods (UPF) at 9 months.

## Abbreviations

The following abbreviations are used in this manuscript:

BMI	Body mass index
IYCF	Infant and Young Child Feeding Indicators
LAZ	Length-for-age z-score
MDD	Minimum dietary diversity
NDSR	Nutrition Data System for Research
NHANES	National Health and Nutrition Examination Survey
UPF	Ultra-processed foods
WAZ	Weight-for-age z-score
WHO	World Health Organization
WLZ	Weight-for-length z-score
