# Supplementary material for: Ultra-Processed Food Intake Is Associated with High Consumption of Added Sugar and Sodium in U.S. Infants
Source: Nutrients. 2026 Sep 13;18(18):2992. doi: 10.3390/nu18182992 (PMC13610061; doi:10.3390/nu18182992)
Supplement: Supplementary file 1 [file nutrients-18-02992-s001.zip › nutrients-4543384-supplementary.pdf]

Table S1: Average dietary intake and growth parameters at 5, 9, and 12 months.

	Baseline (n=45)	9 months (n=85)	12 months (n=84)
Study distribution *			
Study 1	10	25	29
Study 2	35	60	55
Dietary intake †			
Total energy (kcal/day)	55 ± 92	273 ± 198	514 ± 243
Total energy (kcal/kg)	6 ± 7	31 ± 21	56 ± 25
Percent energy Nova 1	48 ± 37	38 ± 22	37 ± 18
Percent energy Nova 2	1 ± 9	1 ± 5	0 ± 2
Percent energy Nova 3	26 ± 36	32 ± 19	25 ± 14
Percent energy Nova 4	27 ± 34	30 ± 19	38 ± 19
Kcal/kg Nova 1	5 ± 13	12 ± 11	22 ± 17
Kcal/kg Nova 2	6 ± 4	3 ± 2	2 ± 3
Kcal/kg Nova 3	5 ± 5	10 ± 7	15 ± 9
Kcal/kg Nova 4	3 ± 3	9 ± 8	20 ± 14
Added sugar (g/day)	0 ± 1	2 ± 4	6 ± 5
Sodium (mg/day)	31 ± 69	246 ± 237	690 ± 372
*Anthropometrics**			
WAZ	-0.47 ± 1.01	-0.27 ± 0.98	-0.10 ± 1.01
LAZ	-0.53 ± 0.99	-0.73 ± 1.14	-0.53 ± 1.12
WLZ	-0.10 ± 0.98	0.23 ± 0.92	0.22 ± 0.90
Weight (kg)	7.03 ± 0.91	8.36 ± 0.99	9.27 ± 1.14
Length (cm)	64.4 ± 2.56	69.37 ± 2.85	73.64 ± 3.06

WAZ, weight-for-age z-score; LAZ, length-for-age z-score, WLZ: weight-for-length z-score.
* Study distribution presented *n* participants.
† Data presented as mean ± SD for all parameters except study distribution (n).

Table S2: Growth outcomes were not significantly associated with UPF intake as percent of total energy in longitudinal mixed effects models.

	Model 1 (% energy) *		
	Estimate	95% CI	p-value†
**WAZ**			
9-month timepoint (5-month ref)	0.38	[0.31, 0.45]	<0.0001
12-month timepoint (5-month ref)	0.52	[0.42, 0.61]	<0.0001
Nova 1 ‡	0.00	[-0.01, 0.01]	0.66
Nova 2 & 3 ‡	0.00	[-0.01, 0.01]	0.70
Nova 4 ‡	0.00	[-0.01, 0.01]	0.89
Total energy intake	0.00	[-0.01, 0.00]	0.18
Study cohort (Study 1 ref)	0.14	[-0.29, 0.56]	0.53
Mode of feeding (EBF ref)	0.16	[0.07, 0.24]	0.0002
**LAZ**			
9 month timepoint (5 month ref)	-0.03	[-0.13, 0.08]	0.61
12 month timepoint (5 month ref)	0.09	[-0.06, 0.24]	0.23
Nova 1 ‡	0.00	[-0.01, 0.02]	0.83
Nova 2 & 3 ‡	0.00	[-0.02, 0.02]	0.95
Nova 4 ‡	-0.01	[-0.02, 0.01]	0.48
Total energy intake	0.00	[0.00, 0.00]	0.65
Study cohort (Study 1 ref)	-0.06	[-0.52, 0.40]	0.80
Mode of feeding (EBF ref)	0.14	[0.01, 0.26]	0.03
**WLZ**			
9 month timepoint (5 month ref)	0.45	[0.34, 0.55]	<0.0001
12 month timepoint (5 month ref)	0.45	[0.30, 0.6]	<0.0001
Nova 1 ‡	0.00	[-0.01, 0.02]	0.72
Nova 2 & 3 ‡	0.00	[-0.02, 0.02]	0.88
Nova 4 ‡	0.00	[-0.01, 0.02]	0.66
Total energy intake	0.00	[0.00, 0.00]	0.11
Study cohort (Study 1 ref)	0.24	[-0.13, 0.61]	0.19
Mode of feeding (EBF ref)	0.09	[-0.03, 0.22]	0.13

UPF, ultra-processed food; CI, confidence interval; WAZ, weight-for-age z-score; EBF, exclusively breastfed; LAZ, length-for-age z-score; WLZ, weight-for-length z-score

* Model 1 reflects includes timepoint, Nova 1, 2&3, and 4 as kcal/kg/d, total calorie intake, study cohort, and mode of feeding. Reference category (where applicable) denoted in first column.

† For all p-values, except interaction terms ($p<0.05$), significance was set at $p<0.006$ after applying Bonferroni correction for multiple testing.

‡ In Model 1, intake of kcal/kg/d was scaled in 10-unit increments, representing the estimated change in outcome per 10 kcal/kg/d increase

Table S3: Dietary intake of UPF was not significantly associated with growth parameters

Outcome	Predictor	β Estimate*	95% CI	p-value†
5 months‡				
WAZ	% kcal Nova 4	0.01	[-0.01, 0.01]	0.73
	kcal/kg Nova 4	0.01	[-0.10, 0.12]	0.84
LAZ	% kcal Nova 4	0.00	[-0.01, 0.01]	0.61
	kcal/kg Nova 4	0.02	[-0.08, 0.12]	0.72
WLZ	% kcal Nova 4	0.00	[-0.01, 0.01]	0.98
	kcal/kg Nova 4	-0.01	[-0.11, 0.10]	0.96
9 months‡				
WAZ	% kcal Nova 4	-0.01	[-0.02, 0.01]	0.61
	kcal/kg Nova 4	0.00	[-0.04, 0.04]	0.93
LAZ	% kcal Nova 4	-0.01	[-0.02, 0.01]	0.39
	kcal/kg Nova 4	-0.01	[-0.05, 0.04]	0.81
WLZ	% kcal Nova 4	0.00	[-0.08, 0.87]	0.94
	kcal/kg Nova 4	0.00	[-0.04, 0.04]	0.93
12 months‡				
WAZ	% kcal Nova 4	0.01	[-0.01, 0.02]	0.43
	kcal/kg Nova 4	0.01	[-0.01, 0.03]	0.58
LAZ	% kcal Nova 4	0.01	[-0.01, 0.02]	0.36
	kcal/kg Nova 4	0.01	[-0.02, 0.03]	0.63
WLZ	% kcal Nova 4	0.00	[-0.01, 0.01]	0.73
	kcal/kg Nova 4	0.00	[-0.01, 0.02]	0.77

UPF, ultra-processed food; CI, confidence interval; WAZ, weight-for-age z-score; LAZ, length-for-age z-score; WLZ, weight-for-length z-score.

* Linear regression models were fit, adjusting for total caloric intake, mode of feeding, and study cohort.

† Significance was determined to be $p<0.006$ using Bonferroni's correction.

‡ Sample sizes: 5 months (baseline, n=45), 9 months (n=85), or 12 months (n=84).

Figure S1: Subject Flowchart of Analytic Sample.

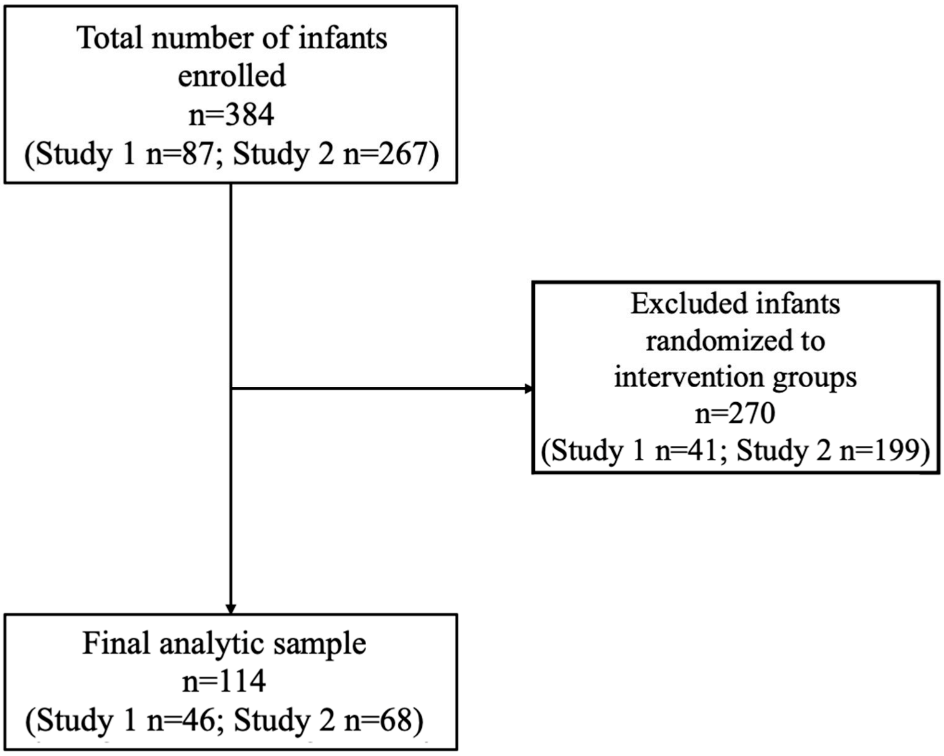

STROBE-nut flow diagram of analytical sample.

Figure S2: Average intake of foods by Nova category as a percent of total energy consumed did not significantly differ at 5, 9, and 12 months.

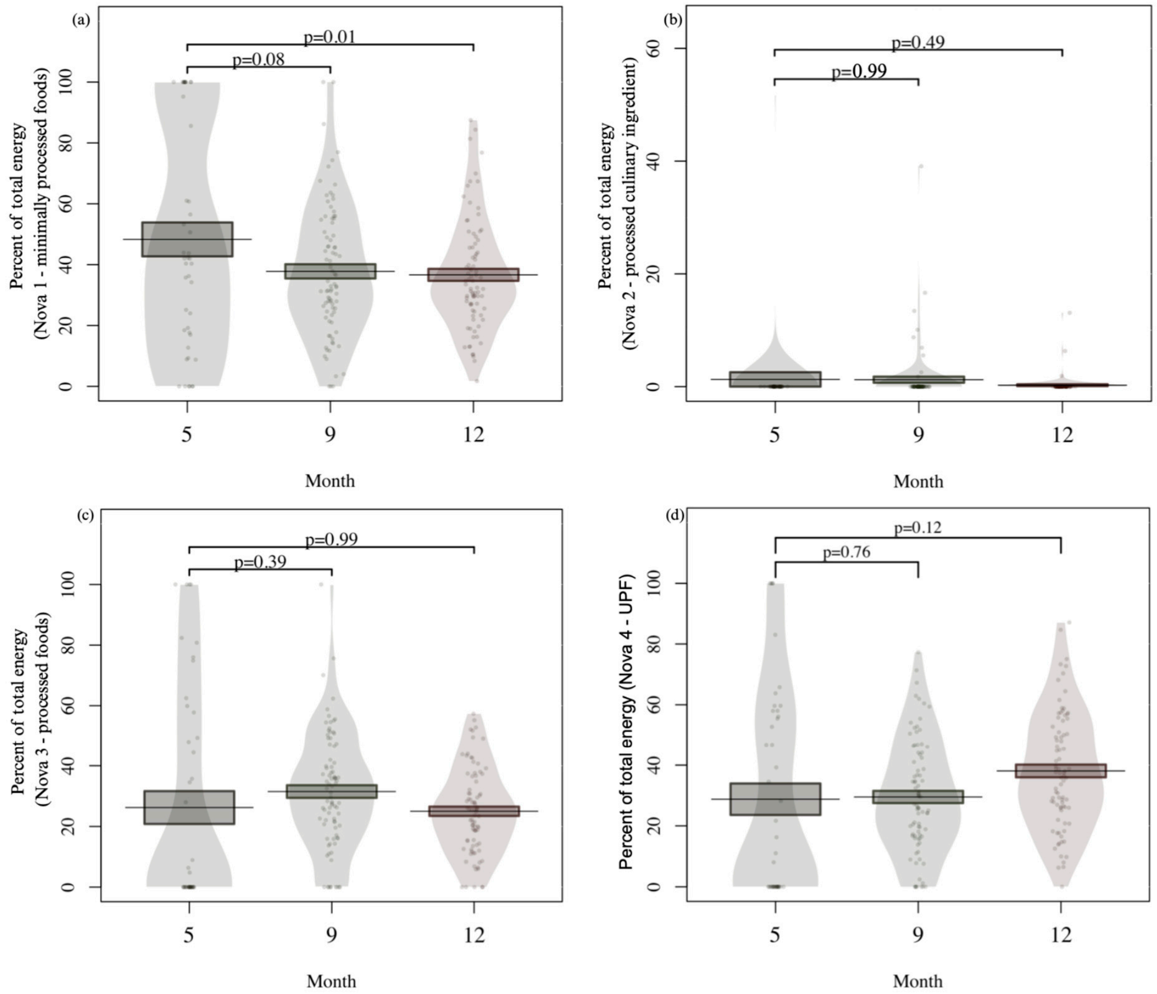

Sample sizes: baseline (n=45); 9 months (n=85); 12 months (n=84). Depicted are a) Nova 1 – minimally processed foods; b) Nova 2 – processed culinary ingredients; c) Nova 3 – processed foods; d) Nova 4 – ultra-processed food (UPF). Changes in Nova category intake over time were assessed using linear mixed effects models with total caloric intake, mode of feeding, and study cohort as covariates and included a random intercept for participant to account for within-subject correlation over time; p-values for differences in intake using pairwise comparisons are shown. Significance was set at $p<0.006$ after Bonferroni's correction.

Figure S3: Natural Log+1 transformed added sugar intake (g) was significantly associated with consumption (kcal/kg/d) of ultra-processed foods (UPF) at 9 months.

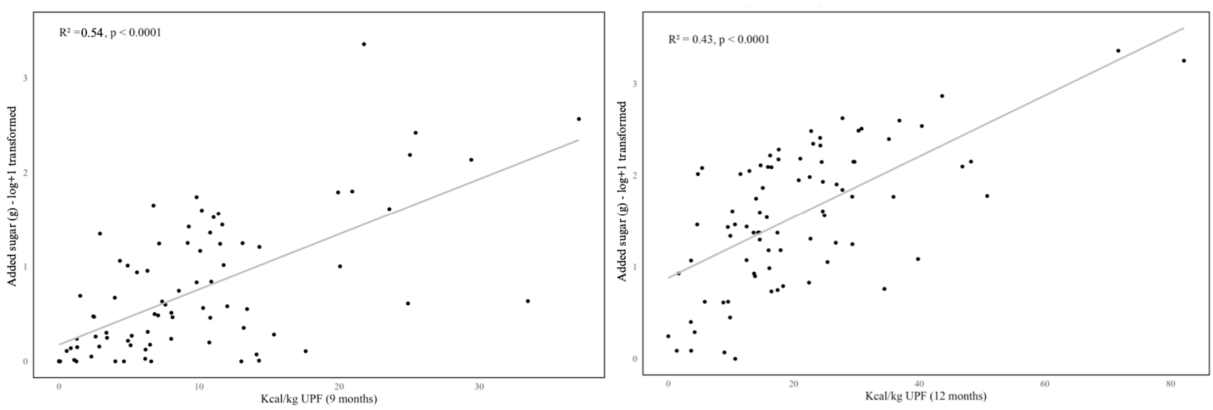

Ultra-processed foods (UPF) were significantly associated with log+1-transformed added sugar intake at 9 (β = 0.06 [0.04, 0.08], p=6.25e-11) and 12 months (β = 0.03 [0.02, 0.04], p=9.89e-11). Data presented is natural log+1 transformed intake from 9 (left) and 12 (right) months of age. Total kcal/kg included as a covariate in the model. R^2 and p-values for the β estimate are presented for each scatterplot. Significance was determined to be $p<0.006$ after applying Bonferroni's correction.
